# Targeting myeloma essential genes using NOT Gated CAR T-cells, a computational approach

**DOI:** 10.1038/s41375-024-02247-1

**Published:** 2024-04-30

**Authors:** Ieuan G. Walker, James P. Roy, Georgina S. F. Anderson, Jose Guerrero Lopez, Michael A. Chapman

**Affiliations:** 1 MRC Toxicology Unit, University of Cambridge, Cambridge, UK; 2 Department of Haematology, University of Cambridge, Cambridge, UK; 3 Addenbrooke’s Hospital, Cambridge Universities Foundation Trust, Cambridge, UK

**Keywords:** Cancer immunotherapy, Myeloma

Despite improvements in therapy, multiple myeloma remains incurable. Chimeric antigen receptor (CAR) T-cells have recently been demonstrated to be highly effective in the disease, but the survival curves are yet to plateau. Finding novel targets and combining targets [1, 2] will improve outcomes. However, CAR T-cells can be activated by minimal levels of target expression, and all existing antigen targets have off-tumour expression; for example, BCMA is expressed in the basal ganglia [3], CD19 in brain mural cells [4], and GPRC5D in the skin and cerebellum [5]. Thus, on-target, off-tumour toxicity is a major problem in target selection and can only become more challenging with combination therapy.

We and others have recently profiled the surface proteome (surfaceome) of myeloma cells [6–8]. In the current study, we began by exploring off-tumour expression of those proteins identified in the myeloma surfaceome. There is a wide range of expression of myeloma surface proteins in healthy tissue (Fig. 1A), and every potential myeloma target is expressed on at least one essential cell type (Fig. 1B). This includes known, or proposed, myeloma immunotherapy targets (Fig. 1C). Thus, no myeloma immunotherapy target is free from the risk of on-target, off-tumour toxicity. One approach to minimise this toxicity is the use of logic gates. NOT-gate CAR T-cells are specifically designed such that their proliferative and cytolytic capacities are downregulated when an inhibitory CAR (iCAR) is bound [9]. The iCAR is selected so that it is absent on the tumour cell, but expressed on any healthy tissue that also expresses the main CAR target [10].

**Fig. 1:**
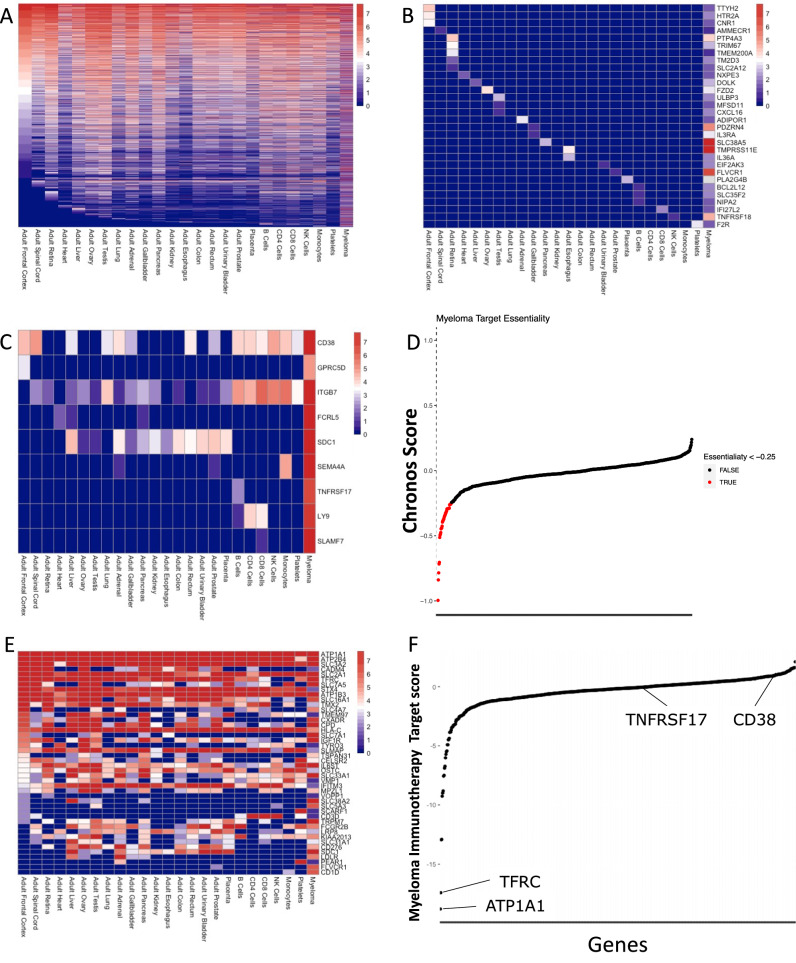
There is no specific cell surface protein target in myeloma. **A** Off-tumour protein expression of all cell surface proteins in myeloma with well-defined extracellular domains (“targetable myeloma proteins”). **B** Healthy tissue protein expression of the thirty targetable myeloma proteins with the lowest off-tumour expression. **C** Off-tumour protein expression of known immunotherapy targets in myeloma. **D** Distribution of Chronos scores for all targetable myeloma proteins. **E** Off-tumour protein expression of the thirty proteins whose expression is most important for myeloma cell viability and/or proliferation (i.e. those with the lowest Chronos scores). **F** Distribution of a vector score encompassing myeloma cell surface expression and myeloma essentiality for all targetable myeloma proteins. The top two hits are indicated, as are the immunotherapy targets, CD38 and TNFRSF17.

We therefore leveraged our knowledge of the myeloma cell surfaceome to explore the landscape of potential NOT-gate targeting in myeloma. To simplify the process for non-bioinformaticians, we encoded our computational pipeline into a publicly available app called **NOT**-gate **A**ntigen and **T**op **E**ssential protein **R**evealer (NOTATER) to help researchers develop rational and safe target antigen combinations in myeloma. At the back end, NOTATER queries three datasets. To determine myeloma cell surface protein expression, it uses our published cell surface proteomic data [1]. For expression on healthy tissues, it queries proteomic data from the Human Proteome Map [11]. Targets that are essential for cancer cell survival are advantageous because they limit the opportunity for antigen escape. Therefore, the third dataset used by **NOTATER** is the Cancer Dependency Map (DepMap) [12]. The latter data employ a Chronos score [13] for each gene, which gives a numerical measure of the loss of viability associated with loss of expression of that gene. Our methodology is included in the Supplemental Materials and our code is publicly available at https://github.com/ieuangw/NOTATER. An online version of the app is available via https://chapman-lab.shinyapps.io/NOTATER.

Herein, we present a case study to identify an effective NOT-gate CAR T-cell strategy to target myeloma. Not only do we describe the potential CAR T-cell targeting landscape of myeloma cells, but we also demonstrate the utility of the NOTATER app. We adopted the following rules:

- The principal target must be expressed on the myeloma cell surface membrane.
- The principal target must have an extracellular domain.
- Loss of the principal target is associated with loss of viability and/or proliferation in myeloma cells.
- The principal target is not expressed on T cells, to prevent T-cell fratricide.
- The iCAR target must not be expressed on myeloma cells.
- The iCAR target must have an extracellular domain.
- The iCAR target must always be co-ordinately expressed with the principal target, except on myeloma cells.

In terms of potential principal targets, there were a total of 5097 plasma membrane proteins in primary myeloma, 1077 of which had a well-defined extracellular domain. Of these, 716 had unique entries in the DepMap dataset. For potential iCAR targets, there were 17,294 proteins in the Human Proteome Map, of which 14,842 were not expressed at all in the plasma membrane of myeloma. 1348 of these had a potentially targetable extracellular domain. There were thus a theoretical 968,032 (716 × 1348) NOT-gate combinations in myeloma. To explore viable combinations, we focused on the most important surface proteins for myeloma cell survival by setting the Chronos score at < −0.25. This identified 45 principal target proteins whose loss is associated with reduced myeloma cell viability and/or proliferation (Fig. 1D). As we had previously shown that there was no completely specific myeloma surface protein (Fig. 1A and B), and as many of the essential proteins were likely to play housekeeping roles, we expected that they would have widespread expression in healthy tissue. Indeed, this was the case (Fig. 1E). However, we reasoned that using a NOT-gate approach could enable these proteins to be used as potential targets. To rank these 45 essential target proteins, we adapted a ranking system similar to that in our previous work [6], combining myeloma cell surface expression and importance for myeloma cell survival into a single score. The two top-ranked target proteins were the cation transport ATPase, ATP1A1, and the transferrin receptor (TFRC) (Fig. 1F). We focused on TFRC because ATP1A1 is a multi-pass protein with only a small extracellular area for scFv binding. Furthermore, TFRC has been previously shown to be a potential target in haematological malignancy [14], although specificity was not addressed in that study.

The NOTATER output from our search is shown in Fig. 2. We filtered for: the most highly expressed proteins in myeloma, requiring median expression above 3.5 arbitrary units (AU; the maximum expression is 5 AU); an extracellular domain size greater than 175 amino acid residues; and a Chronos score of −0.25 or below (Fig. 2A). Filtered genes appear in a drop box (Fig. 2A) from which a principal target gene, in this case TFRC, is selected. The essentiality (Fig. 2B) and expression (Fig. 2C) of this target relative to other myeloma surface proteins are displayed. Feasible iCAR partners for the target are listed in a table (Fig. 2D) and in a heatmap conveying their expression at protein level in healthy tissue and myeloma (Fig. 2E). The top row of this heatmap shows the expression of the principal target in those tissues. Importantly, none of the potential iCAR proteins are expressed in myeloma.

**Fig. 2:**
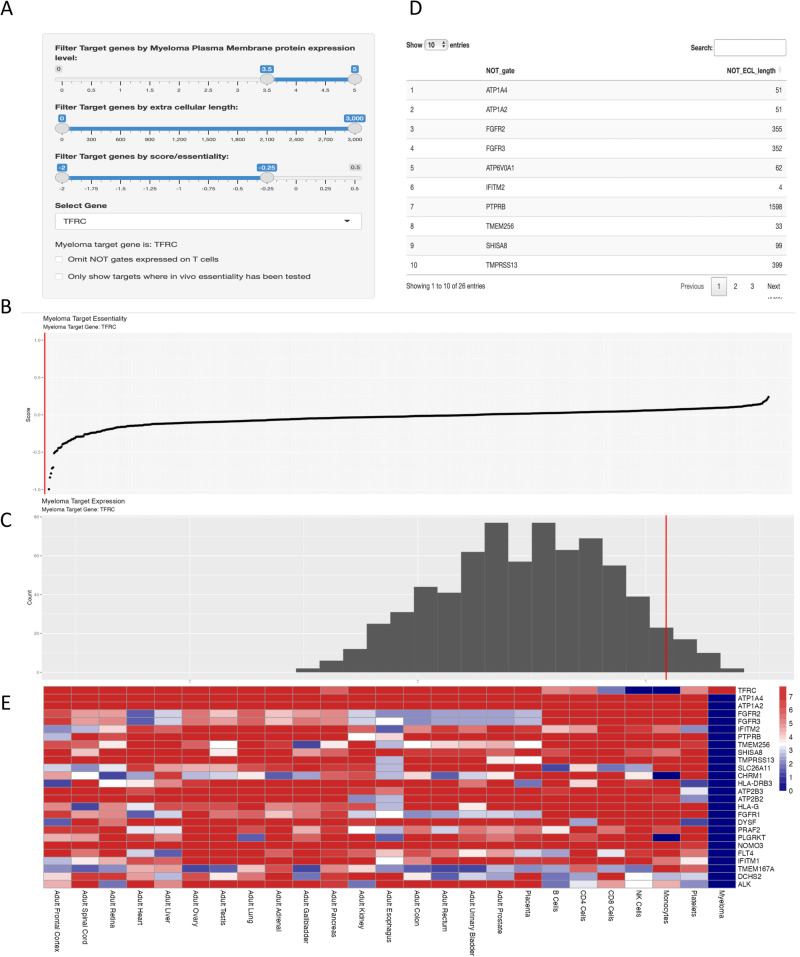
NOTATER app identifies TFRC and iCAR partners for NOT-gate targeting in myeloma. All panels depict screenshots from the app, following selection of TFRC from the dropdown box in A. **A** Filtering panel for level of myeloma cell expression, extracellular domain size, and Chronos score. A dropdown box is used to select the principal cell surface protein target. **B** Essentiality score of the selected principal target protein, shown as a red line relative to the essentiality score of all other targetable myeloma proteins. **C** Myeloma cell surface protein expression of the selected target protein, shown as a red line relative to the essentiality score of all other targetable myeloma proteins. **D** List of potential iCAR proteins that could be used to create a NOT-gate CAR T-cell in conjunction with the principal target for safe targeting. The list can be ordered by protein name or extracellular domain size. **E** Heatmap showing protein expression of the principal target protein (top row) and potential iCAR partners in healthy tissues and myeloma.

From the NOTATER output, it can be seen that TFRC is clearly expressed not only in myeloma cells, but also in the majority of healthy tissues, with the exception of NK-cells and monocytes. However, there are also 26 proteins that are co-expressed with TFRC across those healthy tissues, but not in myeloma (Supplementary Table 1). Their extracellular domain sizes range from 2–2918 amino acids. Excluding the various multi-pass ATPases and proteins with smaller extracellular domains, there remain 13 cell surface proteins which could serve as iCARs in a NOT-gated CAR T-cell against the essential TFRC, whilst preserving specificity. Our data suggest that it is likely that there are also potential iCAR proteins for NOT-gate CAR T-cell targeting of other haematological malignancies with TFRC [14]. To explore the potential for applying NOT-gating to existing known or putative targets, we explored NOT combinations for CD38, TNFRSF17 (BCMA), FCRL5, and SEMA4A (Supplementary Figs. 1–4). There were 31, 307, 104, and 94 possible NOT gate combinations, respectively.

We would note that a limitation of our approach derives from false negatives in DepMap. In some cases, these result from artifacts in CRISPR-Cas9 technology, such as with SEMA4A, where CRISPR-Cas9 targeting results in exon skipping [6]. In others, they may relate to the absence of modelling tumour-microenvironmental interactions in large-scale CRISPR-Cas9 screens. We would therefore advise caution with this aspect of the NOTATER app and recommend running searches with/without essentiality score filters enabled. To partially address the issue of modelling myeloma-bone marrow microenvironmental interactions, we have allowed optional filtering using in vivo CRISPR-Cas9 data from de Matos Simoes and colleagues [15], although the number of surface proteins in that dataset are small.

Herein, we have explored the CAR T-cell targeting space of myeloma. We have shown that there are no truly selective targets in the disease. Interestingly, when we focussed on off-tumour protein expression, the most 30 specific targets do not, in fact, include existing myeloma CAR T-cell targets (Fig. 1C). This may be because myeloma expression and specificity of existing targets were largely determined from transcriptomic datasets, rather than proteomic datasets. This raises the possibility that there are several other myeloma cell surface proteins that could be successfully targeted using conventional CAR T-cells. Nevertheless, our data suggest that CAR T-cell therapy against any myeloma antigen risks on-target, off-tumour effects. Furthermore, if combination targeting employing a mix of CARs is used, these toxicities will only be compounded. B-cell malignancies are amongst the easiest to target with CAR T-cells, as B- or plasma cell aplasia is relatively well-tolerated. On-target, off-tumour toxicity for other cancers, particularly solid organ tumours, makes target selection even more challenging.

NOT-gates represent one tractable approach to reduce or overcome on-target, off-tumour toxicity. This raises two questions. First, should we redesign CAR-T cells against existing targets, such as TNFRSF17, to include NOT-gates? Certainly, it is not clear that the movement disorders associated with TNFRSF17 targeting have been completely overcome. Second, by employing NOT-gates, can we gain a substantial increase in the repertoire of potential targets, e.g. to include proteins that are essential for cancer cell survival? Here we show that even TFRC, a cell surface protein that performs a near-universal housekeeping role, has 13 potential iCARs that could be included in the construct. Thus, NOT-gating is likely to allow the majority of myeloma surface proteins to be used as principal targets.

We also present a simple pipeline that leverages our myeloma cell surface proteomic data, combining it with other publicly available data to select principal CAR T-cell targets and associated iCAR partners. For myeloma, we have created NOTATER, a user-friendly web app which can be used by those without bioinformatics experience to fully explore the landscape of NOT-gate targeting in myeloma. We have also made our code publicly available. With minor modifications, this code could be used to analyse any publicly available or user-defined surfaceome dataset in any cancer to explore novel NOT-gate combinations.

### Supplementary information

Supplemental figures

Supplemental methods

Supplemental table 1

## Data Availability

All code used in the production of this manuscript and the NOTATER app is available at: https://github.com/ieuangw/NOTATER .
