# Supplementary material for: Targeting myeloma essential genes using NOT Gated CAR T-cells, a computational approach
Source: Leukemia. 2024 Apr 30;38(8):1848–52. doi: 10.1038/s41375-024-02247-1 (PMC11286523; doi:10.1038/s41375-024-02247-1)

Supplemental figures

1. NOTATER Output for CD38

2. NOTATER Output for BCMA

3. NOTATER Output for FCRL5

4. NOTATER Output for SEMA4A

Supplementary Figure 1; CD38

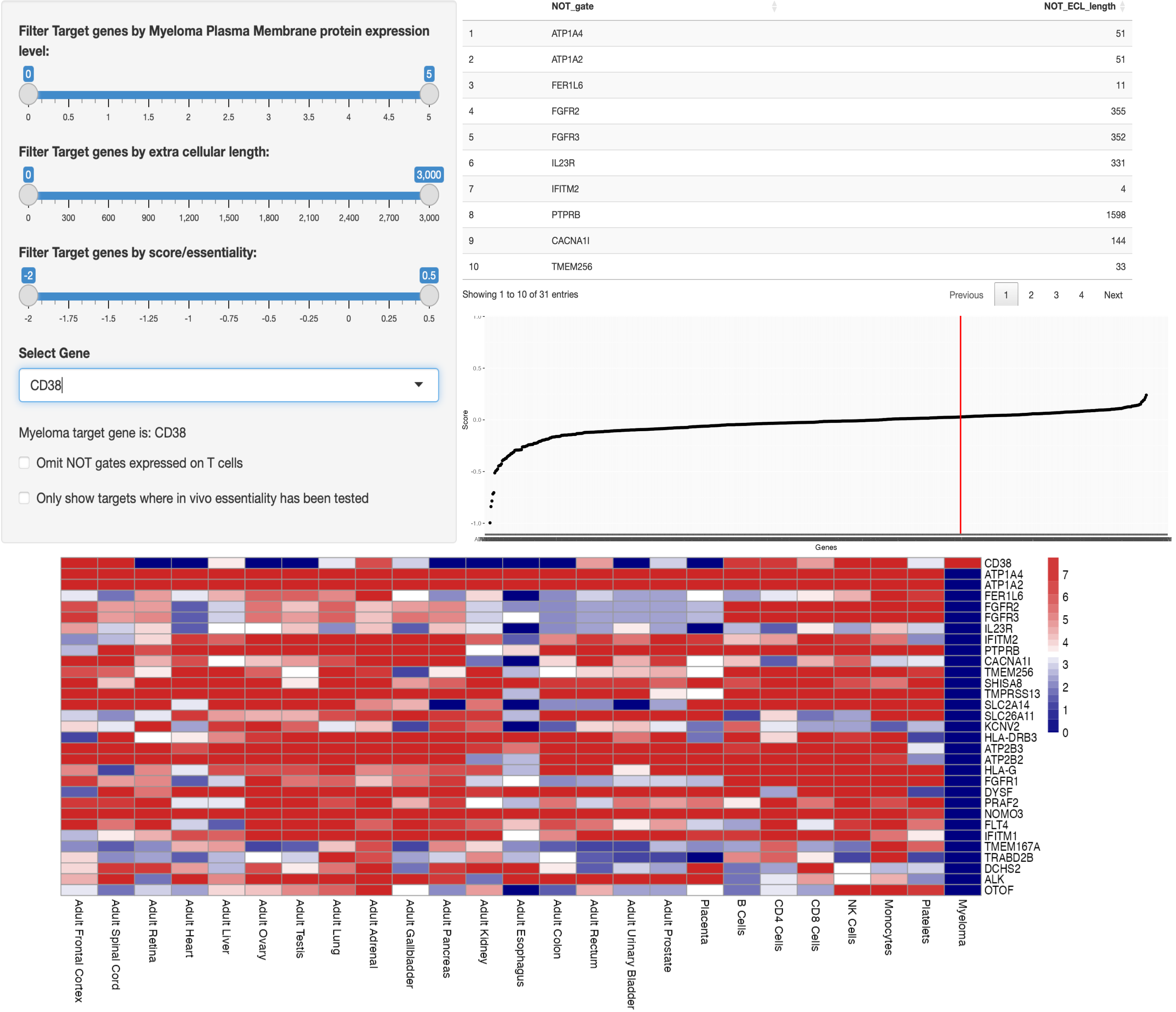

	NOT_gate	NOT_ECL_length
1	ATP1A4	51
2	ATP1A2	51
3	FER1L6	11
4	FGFR2	355
5	FGFR3	352
6	IL23R	331
7	IFITM2	4
8	PTPRB	1598
9	CACNA1I	144
10	TMEM256	33

Supplementary Figure 2: BCMA

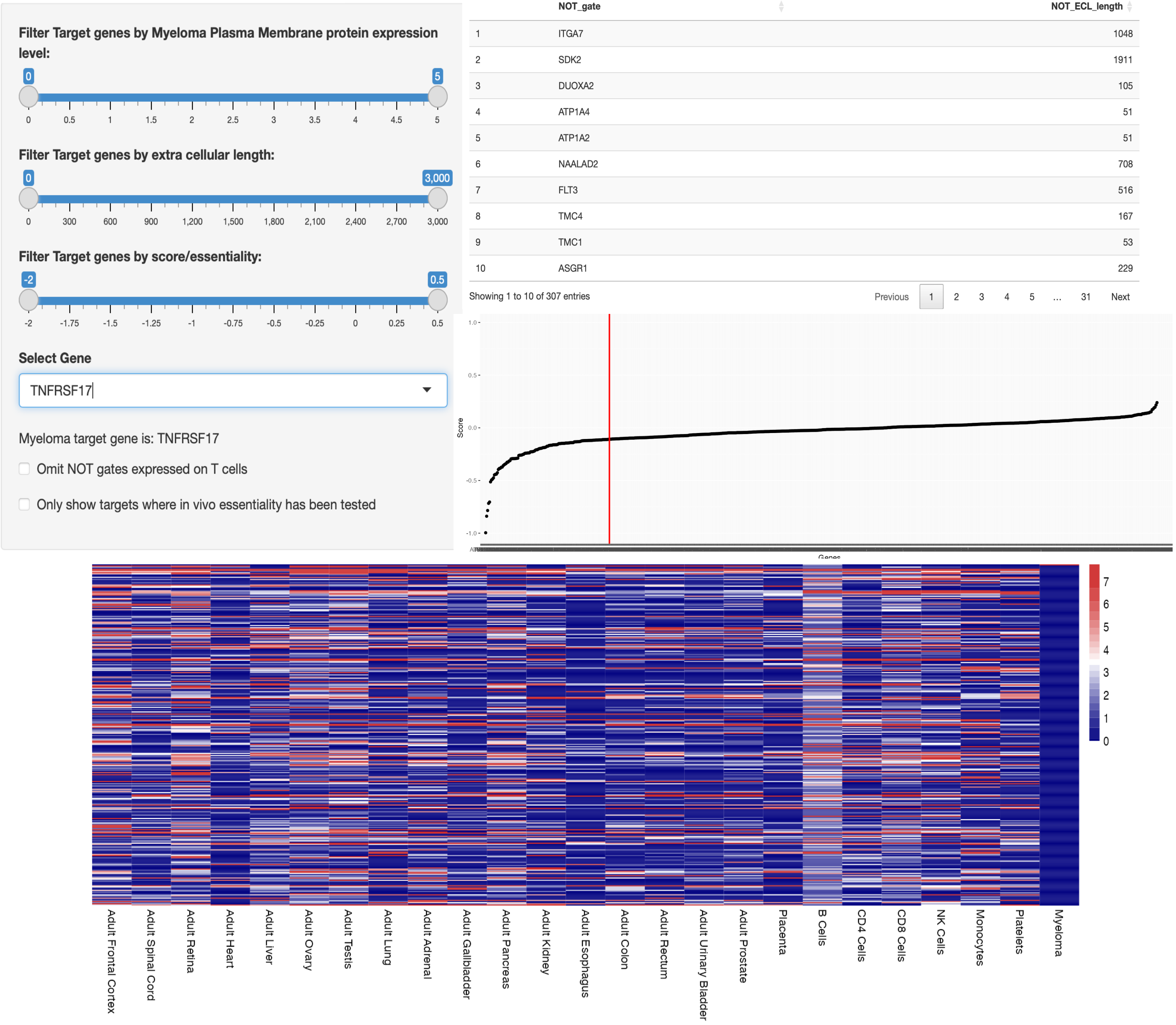

Supplementary Figure 3: FRCL5

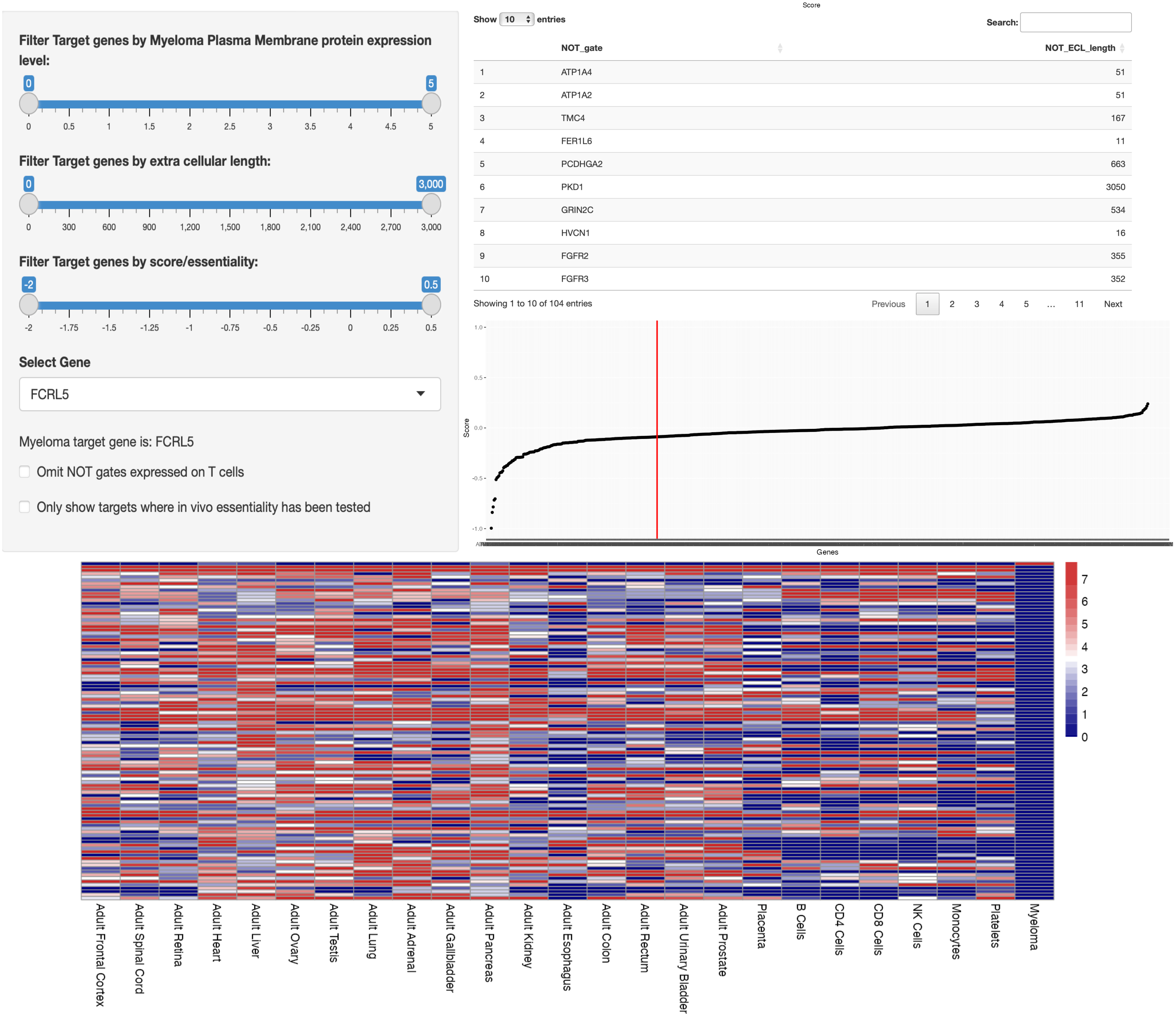

	NOT_gate	NOT_ECL_length
1	ATP1A4	51
2	ATP1A2	51
3	TMC4	167
4	FER1L6	11
5	PCDHGA2	663
6	PKD1	3050
7	GRIN2C	534
8	HVCN1	16
9	FGFR2	355
10	FGFR3	352

Supplementary Figure 4: SEMA4A

Filter Target genes by Myeloma Plasma Membrane protein expression level:

0 – 5

0 0.5 1 1.5 2 2.5 3 3.5 4 4.5 5

Filter Target genes by extra cellular length:

0 – 3,000

0 300 600 900 1,200 1,500 1,800 2,100 2,400 2,700 3,000

Filter Target genes by score/essentiality:

-2 – 0.5

-2 -1.75 -1.5 -1.25 -1 -0.75 -0.5 -0.25 0 0.25 0.5

Select Gene

SEMA4A

Myeloma target gene is: SEMA4A

☐ Omit NOT gates expressed on T cells

☐ Only show targets where in vivo essentiality has been tested

	NOT_gate	NOT_ECL_length
1	ATP1A4	51
2	ATP1A2	51
3	FLT3	516
4	TMC4	167
5	FER1L6	11
6	CLCN1	30
7	HLA-DQB1	197
8	GRIN2C	534
9	HVCN1	16
10	FGFR2	355

Showing 1 to 10 of 94 entries — Previous 1 2 3 4 5 … 10 Next

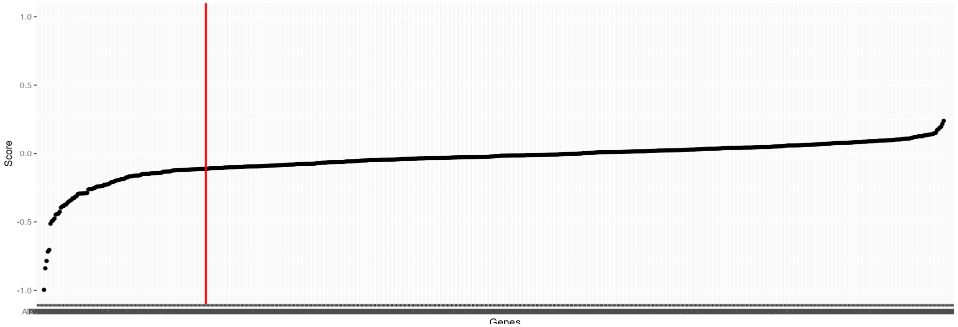

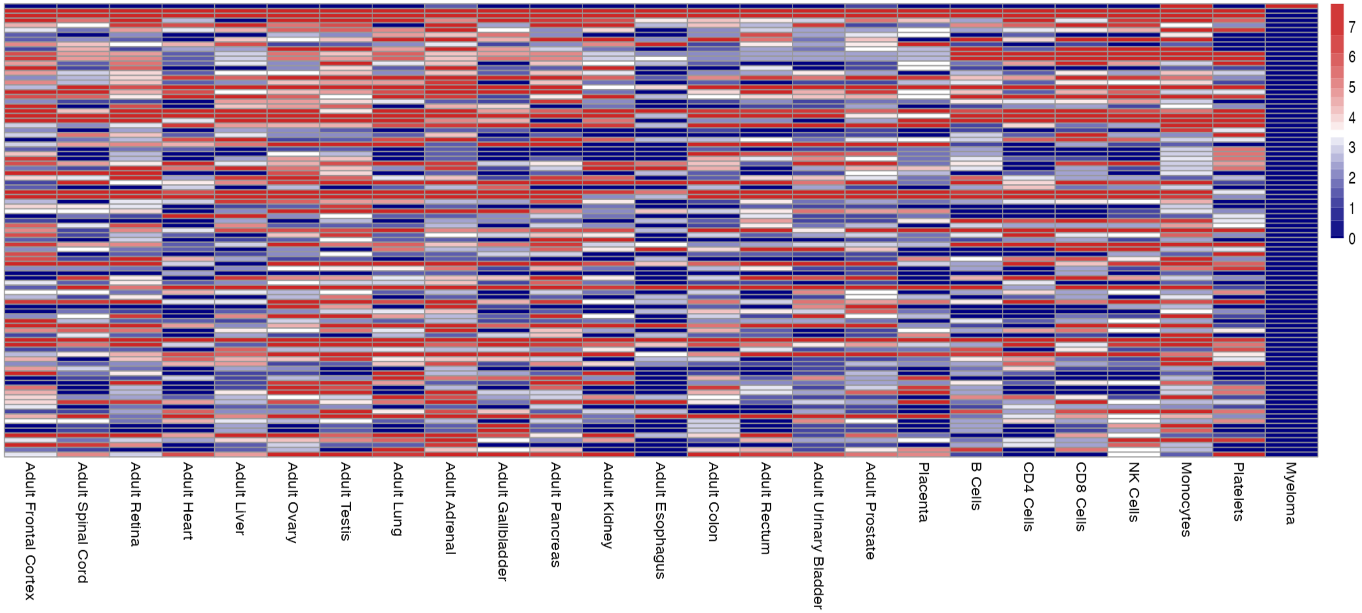

Supplement: Supplementary file 1 — Supplemental figures [file 41375_2024_2247_MOESM1_ESM.pdf]
