# Supplementary material for: Targeting myeloma essential genes using NOT Gated CAR T-cells, a computational approach
Source: Leukemia. 2024 Apr 30;38(8):1848–52. doi: 10.1038/s41375-024-02247-1 (PMC11286523; doi:10.1038/s41375-024-02247-1)
Supplement: Supplementary file 2 — Supplemental methods [file 41375_2024_2247_MOESM2_ESM.pdf]

Supplemental Methods:

Methods.

Problem Formulation.

For optimal selection of myeloma NOT-gated CAR T-Cells we formulated the following criteria.

1) Principal target must be expressed on the MM cell surface.
2) Principal target must have an extracellular domain.
3) Principal target must be essential to MM as defined by CRISPR screening tools.
4) Principal target ideally not expressed on T cells, to prevent fratricide during manufacturing process.
5) NOT target must not be expressed on MM cells.
6) NOT target must have an extracellular domain.
7) NOT target must always be co-ordinately expressed when the principal target is present, except on MM cells.

Selection of Myeloma CAR T Candidates.

Primary MM cell surface proteomic data was obtained from our previous study[1]. Proteins were annotated using the Uniprot database in R[2], and annotation for function, gene name, and extracellular length were obtained. Only proteins that contained an annotated extra-cellular domain were included for considerations as a target.

Annotation of Myeloma Essential genes

Multiple myeloma gene essentiality was obtained from Cancer Dependency Map (DepMap)[3,4]. Chronos score was used to determine gene effect[5]. Thirty-four MM cell lines were identified. Gene essentiality score was determined by the mean score across all cell lines. Only genes which were included in the cell surface protein dataset were considered.

Identifying Target antigen expression and NOT-gate candidates

Proteomic data was obtained from the Human Proteome Map[6]. Expression was defined as any positive expression signal detected in any tissue and was encoded as present or absent. Further annotation was performed using the Uniprot database to denote presence of an extracellular domain and known cellular location. Only proteins known to be cell surface proteins with an extracellular domain were selected as candidates for the NOT-Gate.

Ranking of Target Antigens for Multiple Myeloma

To rank the target antigens, taking account of both expression and essentiality, potential candidates were filtered by the presence of an extracellular domain and the mean log-normalised expression of each protein was multiplied by the effect change score from the DepMap. The most negative scoring proteins were then considered the highest priority immunotherapeutic target.

Selection of Target proteins with NOT-Gate Partners.

Using a custom script in R (version 4.2.2)[7], NOT-gate candidates were selected from the human proteome map, such that the NOT-gate protein was expressed on all tissues that also expressed the main target antigen.

Development of NOTATER

NOTATER was created using the R Shiny framework and relies on dplyr (version 1.1.0)[8] for filtering and ggplot2 (version 3.4.1)[9] for data visualisation. This app has been made freely available for non-commercial use here: https://chapman-lab.shinyapps.io/NOTATER .

All code used in this study was complied in RStudio [10], and is available for review at https://github.com/ieuangw/NOTATER .

Supplemental References

1. Anderson, G. S. F. *et al.* Unbiased cell surface proteomics identifies SEMA4A as an effective immunotherapy target for myeloma. *Blood* 139, 2471–2482 (2022).

2. Carlson, M. & Ramos, M. UniProt.ws: R Interface to UniProt Web Services. R package version 2.38.1. https://bioconductor.org/packages/release/bioc/html/UniProt.ws.html (2022).

3. Tsherniak, A. *et al.* Defining a Cancer Dependency Map. *Cell* 170, 564-576.e16 (2017).

4. Behan, F. M. *et al.* Prioritization of cancer therapeutic targets using CRISPR–Cas9 screens. *Nature* 568, 511–516 (2019).

5. Dempster, J. M. *et al.* Chronos: a cell population dynamics model of CRISPR experiments that improves inference of gene fitness effects. *Genome Biol* 22, 343 (2021).

6. Kim, M.-S. *et al.* A draft map of the human proteome. *Nature* 509, 575–581 (2014).

7. Team, R. C. *R: A language and environment for statistical computing. R Foundation for Statistical Computing,*. (2022).

8. Wickham, H. *et al.* Welcome to the Tidyverse. *J Open Source Softw* 4, 1686 (2019).

9. Wickham, H. ggplot2, Elegant Graphics for Data Analysis. 11–31 (2016) doi:10.1007/978-3-319-24277-4_2.

10. (2020), Rs. T. *RStudio: Integrated Development for R. RStudio*. (Posit Software).
